# Translucency and mechanical behavior of  partially stabilized monolithic zirconia after staining, finishing procedures and artificial aging

**DOI:** 10.1038/s41598-022-20120-y

**Published:** 2022-09-27

**Authors:** Allan Oliveira da Silva, Lívia Fiorin, Adriana Claudia Lapria Faria, Ricardo Faria Ribeiro, Renata Cristina Silveira Rodrigues

**Affiliations:** 1 Department of Dental Materials and Prosthesis, School of Dentistry of Ribeirão Preto, University of São Paulo (USP), Ribeirão Preto, SP Brazil; 2 Department of Dental Materials and Prosthesis, School of Dentistry of Ribeirão Preto, University of São Paulo (USP), Ribeirão Preto, SP Brazil; 3 Department of Dental Materials and Prosthesis, School of Dentistry of Ribeirão Preto, University of São Paulo (USP), Ribeirão Preto, SP Brazil; 4 Department of Dental Materials and Prosthesis, School of Dentistry of Ribeirão Preto, University of São Paulo (USP), Av. do Café, s/n, Ribeirão Preto, SP 14040-904 Brazil

**Keywords:** Biomaterials, Techniques and instrumentation, Materials science, Nanoscience and technology

## Abstract

Partially stabilized zirconia (5Y-PSZ) has been widely used to manufacture indirect monolithic restorations, and the effect of finishing procedures on the optical and mechanical properties of these materials are still unclear. The purpose of this study was to evaluate the effect of staining, polishing and glazing on surface roughness, crystalline phase content, microhardness, fracture toughness, dynamic elastic modulus, three-point flexural strength, strain distribution, color (∆E_00_/∆L/∆a/∆b), and translucency before and after artificial accelerated aging (water spray and ultraviolet) of 5Y-PSZ. Bar-shaped and rectangle-shaped specimens of the 5Y-PSZ were prepared and divided into six groups, according to finishing procedure: GC (control), GS (staining), GG (glazing), GSG (staining and glazing), GP (polishing), GSP (staining and polishing). There was a significant difference between groups for surface roughness (*p* < 0.05), dynamic elastic modulus (*p* = 0.007), microhardness (*p* =  < 0.05), ∆E_00_ (*p* = 0.010), and ∆a (*p* = 0.008). GC presented higher cubic phase content, and the stained groups (GS, GSG and GSP) presented higher monoclinic content. The different finishing procedures affected roughness, dynamic elastic modulus, microhardness, and color of 5Y-PSZ; polishing being the finish that provides minors changes to the 5Y- PSZ. Accelerated artificial aging caused color change, regardless of finishing procedure used.

## Introduction

Dental science is always searching for improvements in optical properties of dental materials. Recently developed monolithic zirconia present lower opacity, greater translucency, and availability of colors for use as a monolithic restorative material^1^. This is attributed to its ability to provide a natural and harmonious smile with other teeth^2,3^.

Polycrystalline yttria-stabilized tetragonal zirconia (3Y-TZP) is the most used in dentistry, but partially (4Y-PSZ and 5Y-PSZ) stabilized zirconia, with higher concentrations of yttrium oxide in its composition when compared to 3Y -TZP, 4 mol% for 4Y-PSZ and 5 mol% for 5Y-PSZ^4,5^, are gaining wider use due to some characteristics. The addition of high concentrations of yttrium oxide promotes an increase in the amount of cubic phase content^5–10^, increasing translucency, since the cubic phase has an index of isotropic refraction, unlike the tetragonal phase^8,10,11^. These modifications are attributed to reduced light scattering that improves the translucency of the material for clinical use^12–14^.

Zirconia is white and requires color adjustment to mimic the appearance of natural teeth^15–18^. There are three techniques available to assign color to zirconia: addition of metal oxides during the manufacture of zirconia blocks, immersion or application of coloring liquids on pre-sintered zirconia, and staining after sintering process^12,19,20^. These methods can affect the crystalline phase content, which can affect the optical and mechanical properties of zirconia^21^.

Polishing and glazing are finishing procedures that improve the aesthetics of zirconia^22^, reducing surface roughness^4,23^, biofilm accumulation^24^, and antagonist wear^25,26^. However, these finishing procedures modify the surface characteristics, and possibly the optical and mechanical properties of the materials, which can reduce the longevity of the indirect restoration^27–32^.

A limited information is available regarding the behavior of 5Y-PSZ after staining and finishing procedures, which are procedures performed routinely in the dental clinic. Therefore, the purpose of this study was to evaluate the effect of staining, polishing, and glazing on surface characteristics and mechanical properties of 5Y-PSZ and the influence of artificial aging on color and translucency. The null hypothesis tested was that staining, polishing, glazing and artificial aging have no influence in evaluated properties of 5Y-PSZ.

## Results

**Figure 1:**
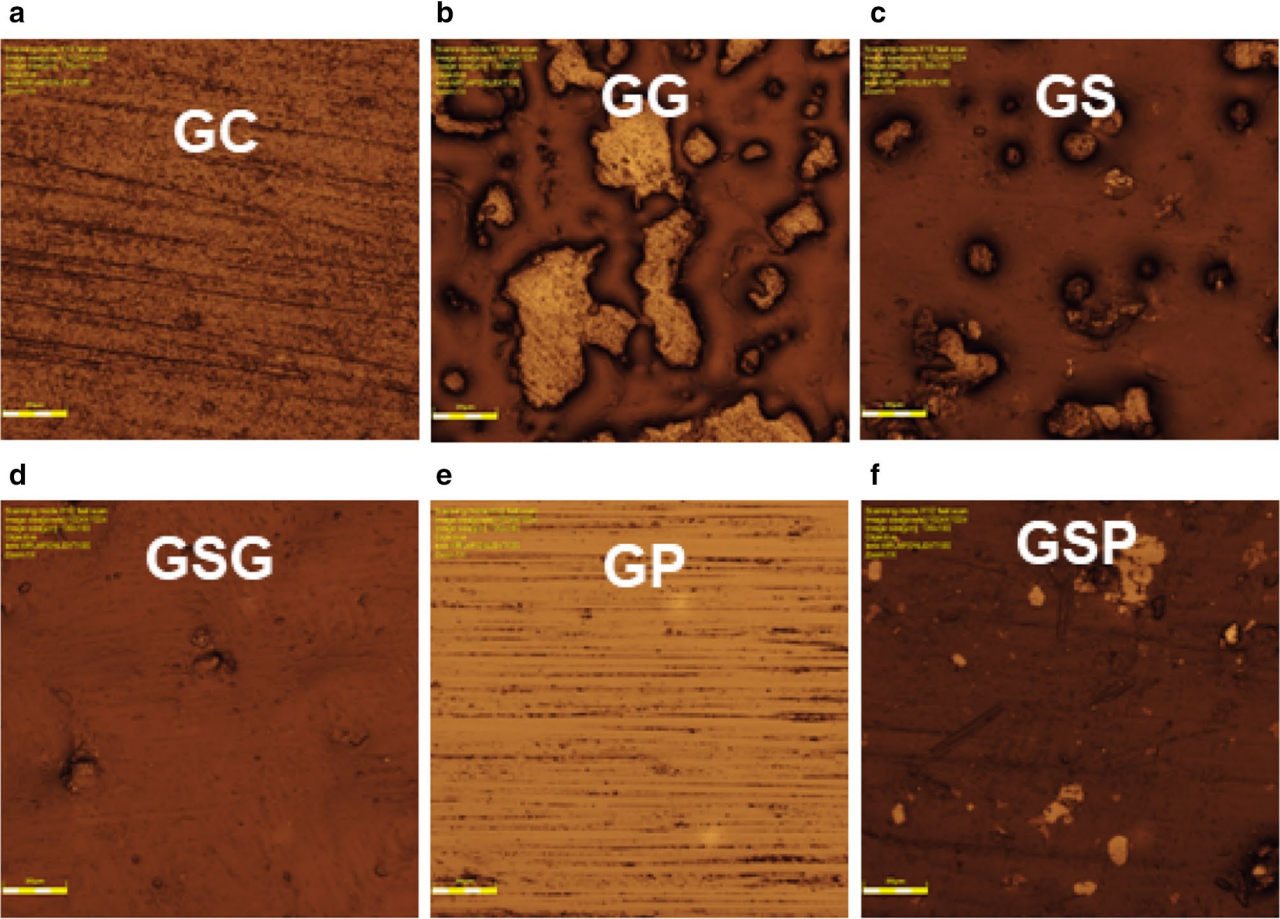
Image captures of the surface morphology of the experimental groups. (**a**) GC (control). (**b**) GG (glazing). (**c**) GS (staining). (**d**) GSG (staining and glazing) (**e**) GP (polishing). (**f**) GSP (staining and polishing).

The surface roughness was different in the experimental groups (Fig. 1). GC and GP showed a regular and similar surface roughness. The combination of staining and finishing procedure (GSG and GSP) provided some defects and an irregular surface, and GG and GS groups showed large irregularities.

A significant difference between groups was found in the roughness (Sa—µm) (*p* =  < 0.05), dynamic elastic modulus (GPa) (*p* = 0.007), and microhardness (KHN) (*p* =  < 0.05). There were no statistical differences between groups in three-point flexural strength (MPa) (*p* = 0.137) and fracture toughness (Kic) (*p* = 0.129). Table 1 presents the results of the surface characteristics and mechanical tests.

**Table 1:** Means and standard deviation and the statistical differences after Tukey's test corresponding to the mechanical properties of the 5Y-PSZ.

Material	Group	Roughness (Sa)	Dynamic elastic modulus (GPa)	Three-point flexural strength (MPa)	Fracture toughness (Kic)	Microhardness (KHN)
		Mean/SD	Mean/SD	Mean/SD	Mean/SD	Mean/SD
5Y-PSZ	GC	1.38 ± 0.12 ^A^	223.78 ± 15.75 ^A^	496.56 ± 129.06 ^A^	1.66 ± 0.55 ^A^	1227.94 ± 79,99 ^A^
	GG	8.09 ± 0.52 ^B^	203.71 ± 7.21 ^AB^	487.75 ± 134.93 ^A^	1.41 ± 0.40 ^A^	711.31 ± 53.17 ^B^
	GS	9.76 ± 0.60 ^C^	193.89 ± 10.33 ^AB^	470.04 ± 142.96 ^A^	1.60 ± 0.47 ^A^	535,28 ± 75.77 ^C^
	GSG	8.94 ± 0.50 ^D^	185.51 ± 10.98 ^B^	482.84 ± 54.73 ^A^	1.52 ± 0.49 ^A^	521.88 ± 48.47 ^C^
	GP	2.40 ± 0.35 ^E^	200.9 ± 19.40 ^AB^	449.24 ± 105.26^A^	1.63 ± 0.43 ^A^	779.72 ± 75.03 ^B^
	GSP	10.12 ± 0.66 ^C^	194.65 ± 20.21 ^B^	589.23 ± 104.57 ^A^	2.05 ± 0.61 ^A^	527.18 ± 34.26 ^C^

*Different letters indicate statistically significant differences between lines.

*SD Standard deviation.

**Figure 2:**
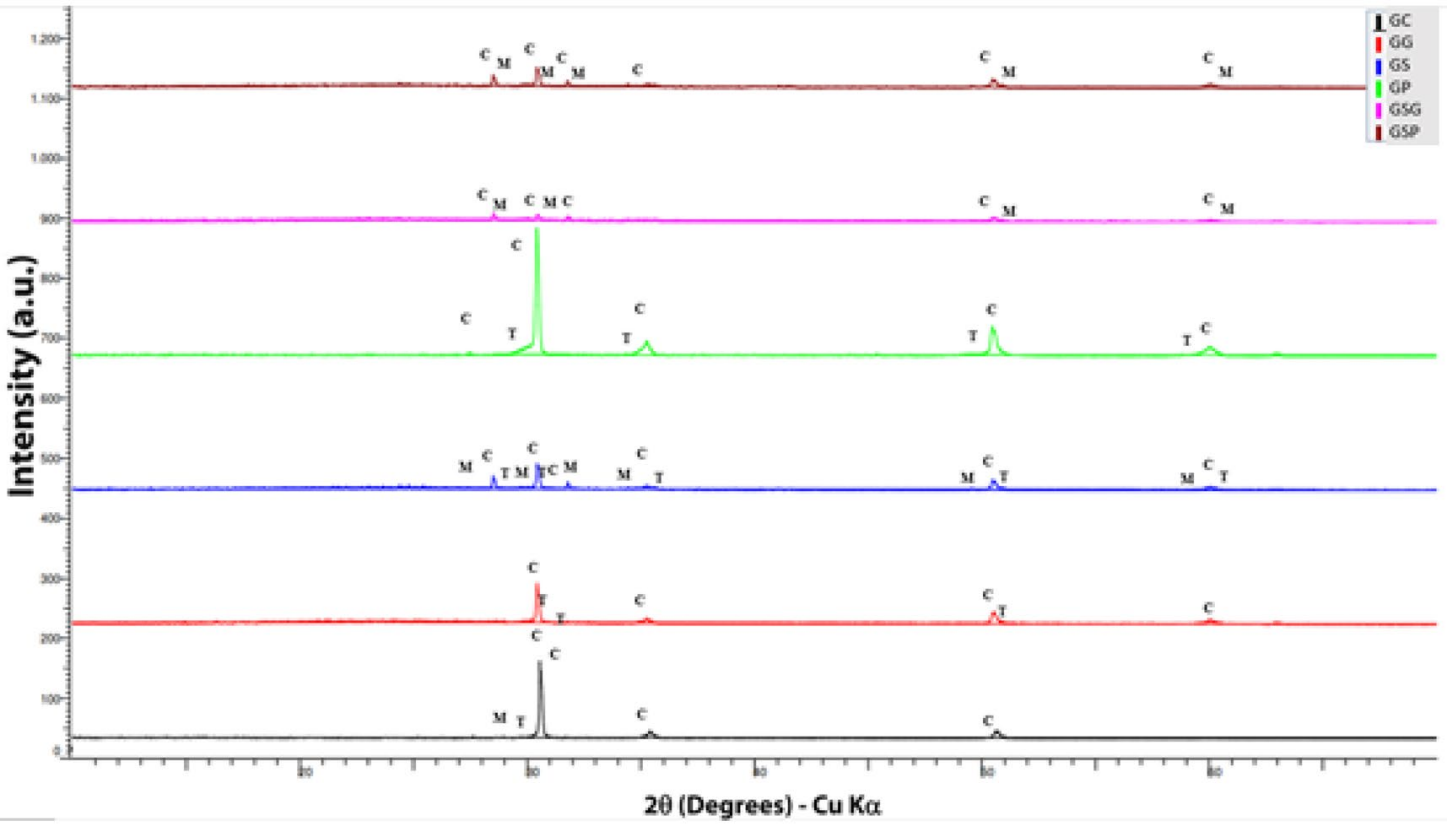
Diffractograms obtained from 5Y-PSZ bars submitted to staining and/or surface finishing procedures.

Figure 2 illustrates the crystalline phase content of 5Y-PSZ obtained by X-ray diffraction and shows that staining increases monoclinic phase content while this phase was not found when only finishing procedures were performed (GG and GP). GC had 95.7% of cubic phase, 1.8% of tetragonal phase, and 2.5% of monoclinic phase. When only glazing was performed (GG), it reduced cubic phase (78.6%), increased tetragonal phase (13.3%) and monoclinic phase was not found. Staining (GS) promoted the most increase of  monoclinic phase content (30.3%), the lower cubic phase content (37.3%) and 24,1% of tetragonal phase. GP had 70.8% of cubic phase, 20.8% of tetragonal phase, and the monoclinic phase was not found. When a combination of staining and finishing procedure (polishing or glazing) was used, tetragonal phase content was not found. GSG presented 60.3% of cubic phase, 29.2% of monoclinic phase, and GSP presented 79.2% of cubic phase and 20.8% of monoclinic phase.

**Figure 3:**
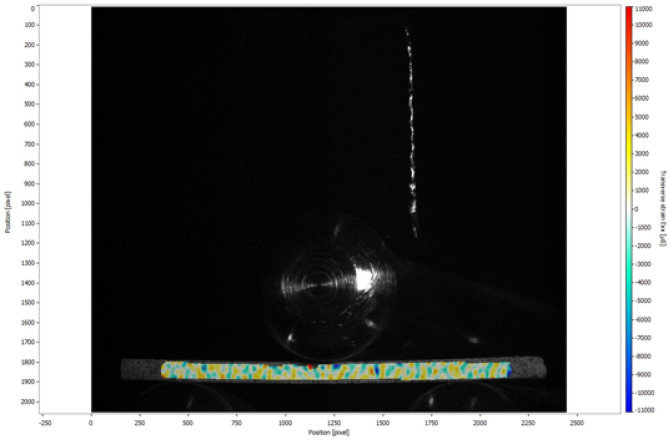
Horizontal strains (Exx) in microstrain (µs) generated during the three-point flexural strength test of the GSG.

**Figure 4:**
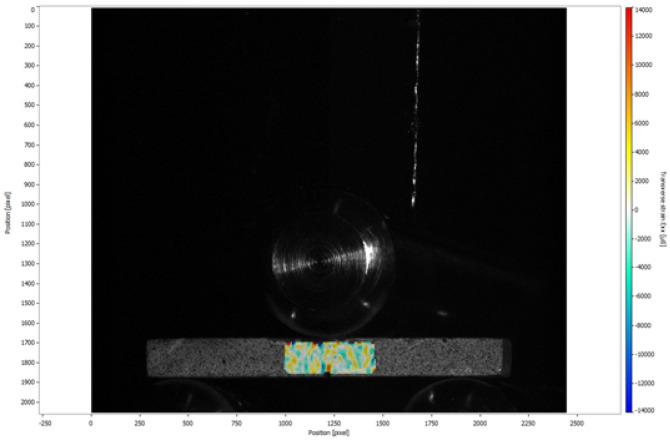
Horizontal strains (Exx) in microstrain (µs) generated during the fracture toughness test of the GSG.

Figures 3 and 4 illustrated the strain maps for 5Y-PSZ, which showed similar behavior for the different groups during the three-point flexural strength and fracture toughness tests. Compressive strains are presented, represented by cold colors, with the white color corresponding to the neutral zone, and the warm colors representing the tensile forces.

**Figure 5:**
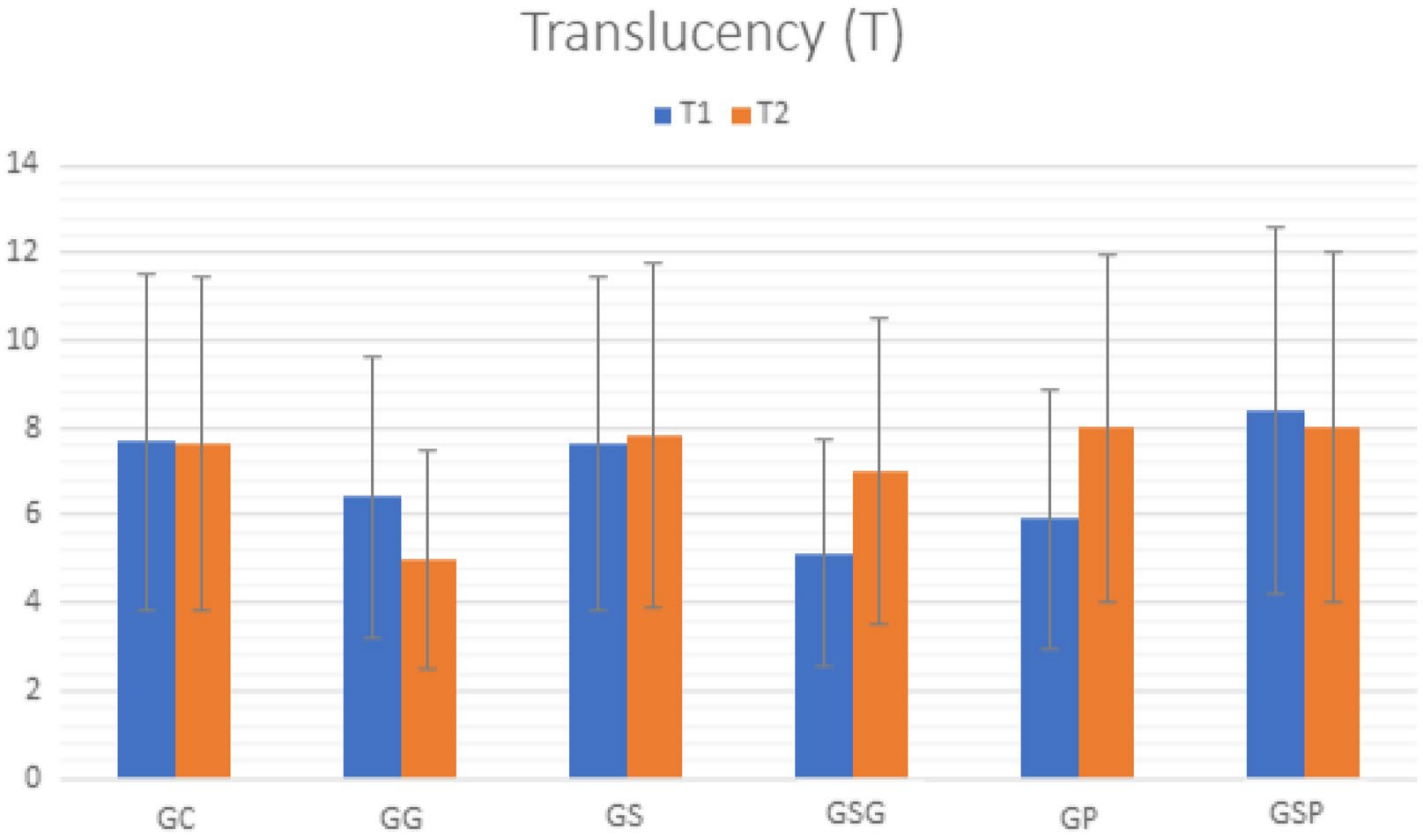
Translucency (T) presented as mean values and standard deviation of 5Y-PSZ groups at T1 and T2.

Table 2 shows the average color variation (∆E, ∆L, ∆a, ∆b) of 5Y-PSZ groups. There was a significant difference between the groups. Figure 5 shows the mean and standard deviation of translucency in each group before (T1) and after the accelerated artificial aging (T2). There was no statistical difference observed in the following comparisons: translucency values at T1 and T2 (*p* = 0.069), groups (*p* = 0.638), and time*group interaction (*p* = 0.533).

**Table 2:** Means and standard deviation and the statistical differences after Tukey's test of color change ∆E_00_, ∆L, ∆a and ∆b for groups at times 1 and 2.

Material	Group	ΔE_00_	ΔL	Δa	Δb
		Mean/SD	Mean/SD	Mean/SD	Mean/SD
	GC	3.80 ± 0.99 ^AB^	2.14 ± 1.07 ^A^	0.27 ± 0.13 ^A^	3.44 ± 1.72 ^A^
	GG	4.34 ± 1.88 ^B^	3.63 ± 1.81 ^A^	0.40 ± 0.20 ^A^	2.84 ± 1.42 ^A^
5Y-PSZ	GS	2.56 ± 1.08 ^A^	2.06 ± 1.03 ^A^	0.81 ± 0.49 ^B^	7.82 ± 3.91 ^A^
	GSG	2.80 ± 0.71^A^	0.93 ± 0.28 ^A^	0.56 ± 0.39 ^AB^	6.98 ± 3.49 ^A^
	GP	3.31 ± 0.97 ^AB^	1.34 ± 0.67 ^A^	0.40 ± 0.20^AB^	7.98 ± 3.99 ^A^
	GSP	3.34 ± 0.52 ^B^	0.45 ± 0.23 ^A^	0.47 ± 0.22 ^AB^	8.01 ± 4.00 ^A^

*Different letters indicate statistically significant differences between lines.

*SD Standard deviation.

## Discussion

The null hypothesis was rejected because staining and finishing procedures affected the surface morphology and roughness, elastic modulus, microhardness, and color of 5Y-PSZ.

5Y-PSZ is indicated to manufacture monolithic crowns in the anterior region^11,33^. Finishing procedures for zirconia restorations, such as polishing and glazing, influence surface characteristics of zirconia, and being indicated for better clinical performance and longevity^22–24^. GC and GP showed a lower surface roughness while the highest roughness occurred in the groups that received glazing and staining. Polishing provides high surface smoothness on 5Y-PSZ^34^ in accordance with Vila-Nova et al.^35^.

The different surface finishing procedures can directly affect 5Y-PSZ microstructure by introducing residual stresses that can impair its mechanical properties^27,29–31^. The lowest microhardness values were associated with groups that received glazing or staining. A higher microhardness was found in GC, GP and GG. This difference is probably attributed to the introduction of a stain/glaze layer at the surface of 5Y-PSZ, whose microhardness is lower, and this layer of glaze does not interact with bulk 5Y-PSZ because of their different microstructure features.

There was no difference among the groups in the three-point flexural strength and fracture toughness test. The results of the present study corroborated Vila Nova et al. (2020)^34^, who obtained mean values of flexural strength of 528 MPa on the 5Y-PSZ. The fracture toughness values of 5Y-PSZ are lower than 3Y-TZP and 4Y-PSZ^35^. This study showed lower toughness values of 5Y-PSZ than other studies with the same methodology, such as: 3.56 MPa^33^, 2.63 MPa^36^ and 2.1 MPa^37^. However, these other studies did not use pre-coloured 5Y-PSZ, which suggests that the introduction of pigments during zirconia block fabrication can lead to a fracture toughness reduction.

The strain distribution during the fracture toughness test showed similar behavior to feldspathic ceramic, lithium disilicate, leucite reinforced ceramic, and nanofilled resin material, and the highest stress concentration occurred on the notch, as the load gradually increased during the test until fracture^38,39^. Staining procedure reduced the dynamic elastic modulus, if compared with GC, that presented highest values. This reduction can probably be caused by metallic salts and oxides present in the stains^18^. There is a similarity between the values of the dynamic modulus of elasticity of 5Y-PSZ with other studies with 4Y-PSZ (210GPa)^40^ and 3Y-TZP (202GPa)^41^.

All groups showed a reduction in the cubic phase content after staining and finishing procedures. Although staining promoted an increase the monoclinic phase content in GS, GSG, and GSP of pre-coloured 5Y-PSZ, the mechanical behavior was not improved. These results are different from Shah et al.^42^, which reported that oxides that give color to 3Y-TZP change the grain size of zirconia and increase the monoclinic phase content, which can improve the mechanical behavior of the material. However, it is necessary to consider differences of 3Y-TZP used in the previous study and 5Y-PSZ of the present study. In addition, the oxides present in the stains can reduce grains, affecting mechanical properties^18^.

Zirconia staining occurs in chromatic reproduction and dental mimicry^21,43–45^. All finishing protocols showed a color change ∆E_00_, perceptible by the human eye with accelerated artificial aging simulating 1 year of clinical service^46–48^. The lower color stability of zirconia is influenced by the properties and qualities of the surface^49^. Differences in color variations may arise from the composition of the material and stain liquids^22^. 5Y-PSZ presents a stable translucency, independent of staining, surface finishing procedures and accelerated artificial aging.

Stains are used in 5Y-PSZ monolithic restorations when color requires adjustment. When stains are necessary, the use of glaze layer to protect stain is important and color change is lower than polishing after stain. However, sometimes color is ideal and stain is not necessary. Then, color stability of polishing is better than glaze. These results are important to guide the dentist in the choice of the ideal finishing procedures in clinical practice.

The study showed that the mechanical behavior of 5Y-PSZ is affected by staining and surface finishing procedures, which are routine laboratory and clinical procedures in the finalization of restorations, exhibiting good results when subjected to the polishing, which is indicated as the best protocol for finishing 5Y-PSZ indirect restorations. Accelerated artificial aging does not affect the translucency of 5Y-PSZ, but changes the color of the material, regardless of the surface finishing procedure. However, the limitations of this study were: design of the specimens was different from dental prostheses, staining was performed in a single layer, using only one color of stain, accelerated artificial aging does not simulate all factors present in the oral cavity, such as pH variation, biofilm formation. In addition, 12 months of clinical service is considered a short period when measuring clinical success of the materials. Clinical studies using staining and surface finishing procedures in 5Y-PSZ indirect restorations are necessary to establish longevity.

## Conclusions

Based on the findings of this in vitro study, the following conclusions were drawn:

- Staining and surface finishing procedures applied on 5Y-PSZ promoted changes in the surface roughness, dynamic elastic modulus, and microhardness.
- Three-point flexural strength and fracture toughness did not differ significantly; polishing promoted minor changes in the mechanical properties of 5Y-PSZ.
- All groups showed crystallographic phase transformation and staining promoted an increase of monoclinic phase content.
- Accelerated artificial aging changes color of 5Y-PSZ, regardless of surface finishing procedure used. Translucency was not affected.

## Methods

Partially stabilized zirconia (5Y-PSZ) blocks (pre-coloured in color A2) (Table 3) were used to manufacture specimens. Bars of two dimensions (30 × 4.4 × 2 mm and 25 × 3 × 4 mm), and rectangle-shaped specimens (5 × 6 × 2 mm) were obtained using a precision saw, with the aid of a diamond disc under water cooling, and manually finished with sandpaper using a sequential granulation of 320, 400, 600, and 1200. Before sintering, the specimens were cleaned or washed in an ultrasonic bath with distilled water, and after sintered in a furnace, according to the manufacturer's recommendations. The specimens were randomly divided into 6 groups: GC (control), GS (staining), GG (glazing), GSG (staining and glazing), GP (polishing), GSP (staining and polishing).

**Table 3:** Commercial name, acronym, composition and manufacturer of the materials used to make the specimens.

Commercial name	Initials	Composition	Fabricator
Ceramill Zolid FX Preshade	5Y-PSZ	ZrO2 + HfO2 + Y2O3: ≥ 99.0, Y2O3: 8.5 – 9.5, HfO2: ≤ 5, Al2O3: ≤ 0.5, Outros óxidos: ≤ 1	Amanngirrbach, Koblach, Áustria

Bar-shaped specimens (30 × 4.4 × 2 mm) (*n* = 60) were used to evaluate surface roughness, crystalline phase content, dynamic elastic modulus, and three-point flexural strength. Other bar-shaped specimens (25 × 3 × 4 mm) (*n* = 60) were used for the fracture toughness test while rectangular-shaped ones (*n* = 66) were subjected to the Knoop microhardness test, color and translucency before and after accelerated artificial aging.

**Figure 6:**
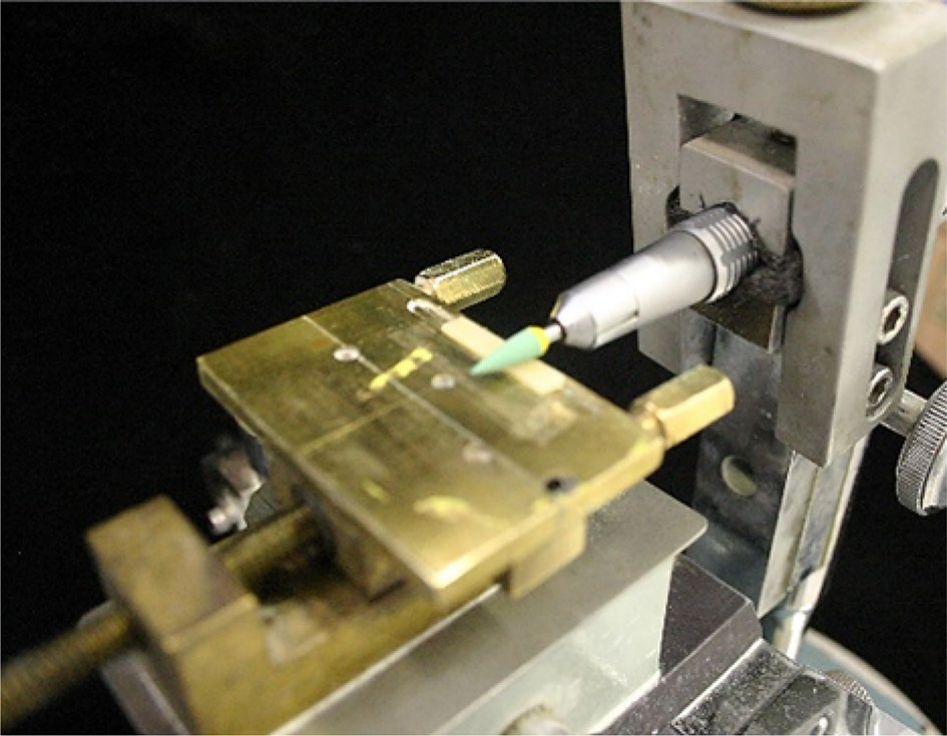
Parallelometer and load control applied during the polishing.

Staining and finishing procedures were performed by a single operator (A.O.S.) after sintering. Glaze (Glaze InSync, InSync, USA) and/or stain (Stain InSync Orange, InSync, USA) was applied using a brush, in a single layer, and fired according to manufacturer’s recommendations. Polishing was performed with diamond polishers in two steps. Initially, samples were polished with a medium-grain diamond polisher (Diacera W16DCmf, Eve Ernst Vetter GmbH, Germany), and after with a fine-grained diamond polisher (Diacera W16DC, Eve Ernst Vetter GmbH, Germany). For this, specimens were positioned in a metallic matrix (Fig. 6). The matrix with specimen and the rotary instrument were positioned in a device that ensures parallelism and standardization of the applied load on all specimens^50^, and polishing was performed using diamond polishers in low speed handpiece at 7000–12,000 rpm.

A confocal laser microscope (LEXT OLS4000, Olympus, Japan) was used to evaluate the surface roughness. A representative image of the surface in each group was chosen based on the repetitive pattern found. The surface roughness (Sa) (*n* = 21) was calculated with software (LEXT 3D Measuring Laser Microscope OLS4000; Olympus) which analyzed the entire scanned surface and calculated a mean roughness. The dynamic elastic modulus was a non-destructive method characterized by the impulse excitation technique, using specific equipment (Sonelastic, ATCP Engenharia Física, Brazil) and software (Sonelastic v. 2.2, ATCP Engenharia Física, Brazil), according to ASTM E-1876. X-ray diffraction (XRD) was performed using a diffractometer (D2 Phaser, Bruker AXS Corporation, USA), and the relative amount of the crystalline phase content was obtained through Rietveld refinement using software (TOPAS V4.2, Bruker AXS Corporation, USA).

The three-point flexural strength test was performed in a universal testing machine (Biopdi, São Carlos, Brazil) using a 100 kgf load cell at a 0.5 mm/min speed, in accordance with ISO 6872^51^. The fracture toughness was calculated using the single-edge V-notch beam method in accordance with ISO 6872:2016^51^. The notch was performed using a diamond disc (0.25 mm rigid sintered diamond, Odontomega, Brazil), having a depth ranging between 0.8 and 1.2 mm. The notches were then finished with a polishing paste (Lunar diamond paste, Odontomega, Brazil) and a razor blade (Navalha, Wilksonnson Sword, United Kingdom). The notches were examined under an optical microscope (S8AP0, Leica, Germany) prior to the test to determine the notching depth between 0.8 and 1.2 mm. The specimens were supported by two rollers with a distance of 20 mm between them. The notched surface was positioned downwards, and loaded under 0.5 mm/min speed in an universal testing machine until the fracture. The digital image correlation was used to qualitatively analyze the strain distribution in the 5Y-PSZ bars during the three-point flexural strength and fracture toughness tests. The Knoop microhardness was evaluated using a microhardness tester (HMV-2, Shimadzu Corp., Japan) under a load of 3 N for 15 s (five indentation were applied for each specimen at five different locations)^52^. Color and translucency were evaluated using a spectrophotometer (Delta Vista 2.0, Delta Color, Brazil) at room temperature according to the formulas described by Sharma et al. and Nassary et al.^33,53^. Color and translucency were evaluated before (T1) and after (T2) artificial aging. Artificial aging was performed using the Accelerated Aging System for C-UV non-metallics (Conexim Matérias Primas Ltda, São Paulo, Brazil), with condensation water spray with saturated air-vapor mixture and UV-B light with radiation concentrated between 280 and 320 nm. The program consisted of 4 h exposure to UV-B light at 50 °C and 4 h condensation at 50 °C for a period of 300 h. This aging procedure corresponds to 1 year of clinical service^48–50^.

Statistical analyses were performed using IBM SPSS statistics software (20.0, IBM, USA), after the Shapiro–Wilk normality test, data from all assays presented normal distribution. The surface characteristics, mechanical properties and color were evaluated by one-way analysis of variance (ANOVA) and post-hoc Tukey test (α = 5%). Translucency was evaluated by the linear model of repeated measures and Bonferroni's test.

## Data Availability

Data supporting the results of this study are available in the article and can be requested from the corresponding author.
